# Indications and Diagnostic Yield of Paraneoplastic and Autoimmune Encephalitis Antibody Testing: A Retrospective Cohort Study

**DOI:** 10.1002/brb3.70779

**Published:** 2025-08-22

**Authors:** Hannah Ford, Nabil Seery, Tracie Tan, Logan Gardner, Jeffrey Box, Robb Wesselingh, Julian Bosco, Mastura Monif

**Affiliations:** ^1^ Department of MS and Neuroimmunology Alfred Hospital Melbourne Victoria Australia; ^2^ Department of Pathology Alfred Hospital Melbourne Victoria Australia; ^3^ Asthma, Allergy and Clinical Immunology Alfred Hospital Melbourne Victoria Australia; ^4^ Department of Neuroscience, Central Clinical School Monash University Melbourne Victoria Australia

**Keywords:** autoantibodies, autoimmune encephalitis, neurologic autoimmune diseases, paraneoplastic neurologic syndromes

## Abstract

**Background:**

Autoimmune encephalitis (AE) and paraneoplastic syndromes (PNS) are rare disorders with distinct phenotypes. They are increasingly considered in patients with diverse neurologic symptoms, but the clinical context remains critical for diagnosis. Commercial diagnostic assays for AE and PNS are embedded as available tests, however the diagnostic yield and clinical impact of autoantibody testing remain uncertain.

**Methods:**

This single‐center study analyzed all cerebrospinal fluid (CSF) and serum neuronal cell‐surface antigen antibody (NCSAA) and intracellular neuronal antigen antibody (INAA) tests requested as part of routine care between 2019 and 2022. Data was collected retrospectively on clinical indications, supportive investigations, diagnosis, and management.

**Results:**

2161 antibody tests (1371 serum, 790 CSF) from 491 patients were reviewed. The most frequent testing indications were cognitive (26.5%, 130) and psychiatric (20.6%, 101) syndromes, 56.9% (74) and 18.8% (19) of which had associated focal neurology/seizures, respectively. Other common indications included neuropathy, seizures, movement disorders, ataxia, visual symptoms, myelitis, and motor neuron syndromes. Only 1.6% (34) of all autoantibody tests were positive, 38.2% of which were true positives (seven NCSAA, six INAA) and 61.8% (21) were false positives (one NCSAA, twenty INAA). Most false positives (95.2%, 20) were tested in serum only. 0.8% (4) of patients were diagnosed with seropositive AE (LGI1, NMDAR) and 0.8% (4) with INAA‐associated syndromes (CRMP5, Yo, GAD65). All had focal neurology/seizures or supportive paraclinical investigations. 98.4% (2127) of antibody tests were negative, 99.1% (2107) of which were true negatives and 0.9% (20) were false negatives (19 seronegative AE, one seronegative PNS). 24% (118) of patients received immunosuppression, 93.2% (110) of whom were antibody negative.

**Conclusions:**

Our findings demonstrate that indications for autoantibody testing are heterogenous, with a low true positive yield and frequent INAA false positives. This underscores the importance of clinical phenotyping and supportive investigations to prevent inappropriate widespread autoantibody testing.

## Graphical abstract

This single center retrospective study analyzed testing indications and yield of neuronal cell‐surface antigen antibody (NCSAA) and intracellular neuronal antigen antibody (INAA) testing for autoimmune encephalitis (AE) and paraneoplastic syndromes (PNS). Cognitive and psychiatric symptoms were the most common testing indications. The yield of positive test results was low (1.6%), and 68.3% of positive results were false. < 1% of patients was diagnosed with seropositive AE or PNS. Clinical phenotyping with supportive investigations should be prioritized prior to autoantibody testing.

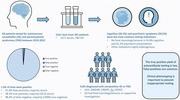

## 1 Introduction

The autoimmune encephalitides are rare immune‐mediated inflammatory brain disorders classified broadly into two groups—the first involving antibodies targeting neuronal cell surface antigens and the second with antibodies against intracellular neuronal antigens (Lancaster and Dalmau 2012). In the former group, neuronal cell surface antigen antibodies (NCSAA) are directly pathogenic due to the binding of extra‐cellular cell surface proteins leading to neuronal dysfunction (Dalmau et al. 2017, Leypoldt et al. 2013). In the latter, intracellular neuronal antigen antibodies (INAA) are not considered directly pathogenic and represent an immunological epiphenomenon in the setting of cytotoxic T‐cell mediated inflammation and neuronal loss (Bien et al. 2012, Marsili et al. 2023).

The discovery of anti‐N‐methyl‐D‐aspartate receptor (NMDAR) antibodies causing a specific syndrome characterized by psychosis, seizures, and movement disorders (Dalmau et al. 2008) led to rapid growth in autoimmune encephalitis (AE) research. Since this seminal publication there has been an exponential increase in the discovery of autoantibodies associated with central nervous system (CNS) inflammatory conditions. Presentations of AE and paraneoplastic syndromes (PNS) are clinically heterogenous, often involving polysymptomatic neurology and/or profound neuropsychiatric disturbances (Graus et al. 2016, Uy et al. 2021, Gilligan et al. 2023). EEG, MRI, and CSF evaluation are key diagnostic tools, alongside paired serum and CSF autoantibody testing (Abboud et al. 2021, Binks et al. 2022, Irani 2024). Although the spectrum of presentations is broad, distinct clinical phenotypes exist for individual AE and PNS subtypes (Ramanathan et al. 2021, Devine et al. 2021, Graus et al. 2021, Dalmau and Graus 2018). Previous reviews have emphasized a phenotype‐based approach to autoantibody testing and provided guidance on which autoantibody profiles should be ordered based on the neurologic syndrome (Gilligan et al. 2024, Graus 2024).

AE and PNS are rare conditions (Dubey et al. 2018, Vogrig et al. 2020). Despite this, these diagnoses are increasingly listed as differentials for patients with a wide range of neurologic, psychiatric and cognitive symptoms. NCSAA and INAA tests are often grouped together and ordered for patients whose clinical syndromes do not align with the phenotypes associated with all the tested autoantibodies (Ebright et al. 2018). Recent literature has highlighted the potential harms of widespread testing in patients with a low pre‐test probability, including false positives, misdiagnosis, inappropriate immunotherapy, and deferral of accurate treatment (Ebright et al. 2018, Flanagan et al. 2023, Zidan et al. 2019, Budhram et al. 2020). Autoantibody tests are costly and labor intensive (Zidan et al. 2019, Deng et al. 2022, Sharp et al. 2021). The yield of testing for AE and PNS has not been clearly defined. This single‐center retrospective cohort study aimed to review the clinical indications and yield of autoantibody testing based on current practices.

## 2 Methods

### 2.1 Study Population

This study was approved by the Alfred Hospital Ethics Committee (437/23). Results from all NCSAA and INAA tests requested from Alfred Health between 2019 and 2022 were collected. Alfred Health is a tertiary hospital in Melbourne, Australia, with a catchment covering 750, 000 people. (Health 2023) Samples sent from external sites were not collected. Results were excluded if testing was sent as part of an Electronic Medical Records (EMR) training program or from external practitioners without clinical information on EMR. Results of NMDAR, α‐amino‐3‐hydroxy‐5‐methyl‐4‐isoxazolepropionic acid receptor types 1 and 2 (AMPAR1 and AMPAR2), gamma‐aminobutyric acid A and B (GABA‐A, GABA‐B), leucine‐rich glioma inactivated 1 (LGI1), contactin‐associated protein‐like 2 (Caspr2), IgLON5 and INAA “panel” antibody tests sent in cerebrospinal fluid (CSF) and serum were searched for in EMR. INAA panels included Hu, Yo, Purkinje Cell, Ri, GAD65, Amphiphysin, PNMA2, SOX1, Titin, Recoverin, CRMP5 and Zic4 antibodies. Patient data was retrospectively analyzed from EMR, including clinical indication for testing, supportive investigations (CSF, EEG, MRI brain), final diagnosis, and management.

### 2.2 Antibody Testing

All INAA and NCSAA testing was conducted using standardized protocols at external, centralized reference diagnostic laboratories in Australia across Melbourne (Victoria), Sydney (New South Wales) and Brisbane (Queensland). NCSAA testing was performed using indirect immunofluorescence (IIFT). NMDAR antibodies were detected with a commercial assay containing four biochips of primate cerebellum, primate hippocampus, fixed NR1‐transfected human embryonic kidney 293 (HEK293) cells, and fixed non‐transfected control HEK293 cells (IIFT: Glutamate Receptor Mosaic 3, Euroimmun, Lübeck, Germany). Testing for LGI1, CASPR2, AMPAR1 and 2, and GABA‐B antibodies utilized a commercial assay with biochips of fixed HEK293 cells transfected with LGI1, CASPR2, GABA‐B, AMPAR1, and AMPAR2 (IIFT: Autoimmune Encephalitis Mosaic 6, Euroimmun, Lübeck, Germany). IgLON5 antibodies were screened for using a commercial fixed slide of primate cerebrum, primate cerebellum and murine stomach (NOVA Lite Monkey Cerebellum/Cerebrum and Mouse Stomach Slide Pack, Inova Diagnostics, San Diego, CA, USA), and then confirmed by IIFT using a commercial assay with biochips of fixed HEK293 cells transfected with IgLON5 (IIFT: IgLON family member 5, Euroimmun, Lübeck, Germany).

Serum INAA samples were screened by IIFT using a commercial fixed slide of primate cerebellum, primate cerebrum and murine stomach (NOVA Lite Monkey Cerebellum/Cerebrum and Mouse Stomach Slide Pack, Inova Diagnostics, San Diego, CA, USA). If there was a suspicious immunofluorescence pattern, confirmatory line blot testing was performed using a paraneoplastic neuronal for antibodies against Hu, Yo, Purkinje Cell, Ri, GAD65, Amphiphysin, PNMA2, SOX1, Titin, Recoverin, CRMP5, and Zic4 (EUROLINE: Paraneoplastic Neurologic Syndromes ‐ 12 Ag, Euroimmun, Lübeck, Germany). CSF INAA samples were screened by IIFT only using the tissue‐based assay (TBA) described above; line blot testing was not performed.

### 2.3 Statistical Methods

Descriptive statistics were used to summarize patient demographics, test indications, and investigation findings.

### 2.4 Interpretation of Autoantibody Test Results

A positive NCSAA was defined as a true positive if the patient met the 2016 diagnostic criteria for possible or definite AE (Graus et al. 2016). A positive INAA was defined as a true positive if associated with a known neurologic phenotype as per 2021 PNS diagnostic criteria (Graus et al. 2021). False positive results were defined as positive NCSAA or INAA in patients not meeting either of the above criteria. True negatives were defined as negative NCSAA or INAA in patients not meeting either of the above criteria. False negatives were defined as negative autoantibody results in patients meeting diagnostic criteria for possible seronegative AE (Graus et al. 2016) or probable PNS (PNS‐Care Score of ≥ 6) without another explanation (Graus et al. 2021).

## 3 Results

A total of 2182 CSF and serum tests were sent from 504 patients between 2019 and 2022. Twenty one tests from 13 patients were excluded due to inadequate clinical information, leaving 2161 tests (1371 serum, 790 CSF) from 491 patients for analysis. 822 tests (38%) were sent in serum only and 241 (11.2%) in CSF only. The remaining 1098 CSF and serum tests were sent as paired specimens (50.8%).

### 3.1 Demographics

Patient demographics are summarized in table 1. The median age was 58. Gender split was similar (female to male ratio 1.01:1). Most patients were tested from an inpatient setting (343, 69.9%). Neurology, psychiatry, and general medicine ordered the majority of autoantibodies.

**TABLE 1:** Patient demographics: patient characteristics and respective medical specialties responsible for antibody testing.

Category		No.
**Patients**		491
**Tests**		2161
	*Serum*	1371
	*CSF*	790
**Median age (range)**		58 (14 – 90)
**Biological sex**		
	*Male*	244
	*Female*	247
**Setting**		
	*Inpatient*	343
	*Outpatient*	148
**Specialty**		
	*Neurology*	373
	*Psychiatry*	54
	*General Medicine*	22
	*Rheumatology*	7
	*ICU*	6
	*Hematology*	5
	*Cardiology*	3
	*Geriatrics*	3
	*Oncology*	3
	*Rehab*	2
	*Emergency Medicine*	2
	*Ophthalmology*	1
	*Respiratory*	1
	*Renal*	1
	*Immunology*	1

Abbreviations: CSF: cerebrospinal fluid; ICU: intensive care unit.

### 3.2 Clinical Indications

Clinical indications for antibody testing and supportive investigations are summarized in table 2. The most common indications for testing were cognitive and psychiatric syndromes. 130 patients (26%) presented with cognitive impairment and 101 (20.6%) with psychiatric symptoms, 73 (56.2%) and 19 (18.8%) of which had associated seizures/focal neurology respectively. Other common indications included peripheral neuropathy (65, 13.2%), seizures/epilepsy (53, 10.8%), movement disorders (30, 6.1%), ataxia (25, 5.1%), visual symptoms (17, 3.7%), myelitis (11, 2.2%) and motor neuron syndromes (10, 2%). Testing indications for the remaining 49 patients were heterogenous or unknown. The following were listed as testing indications (≤ 5 patients each)– peripheral nerve hyperexcitability, myopathy, dysautonomia, unexplained fever, bulbar palsy, sleep disorder, cranial neuropathies, vestibular syndrome without ataxia, monitoring of previous AE, chronic pain, ophthalmoplegia, hemiparesis, focal sensory disturbances, vasculitis, neuromuscular disorders, or headache. Three patients had no clear testing indication.

**TABLE 2:** Clinical indications and supportive investigations: indications for ordering antibody tests and associated abnormal ancillary investigation results.

Indication	No. (%)	Abnormal MRI	Abnormal EEG	Abnormal CSF
**Isolated cognitive impairment**	57 (11.6)	12	19	26
**Cognitive impairment + seizures/focal neurology**	74 (15.1)	35	53	39
**Isolated psychiatric syndrome**	82 (16.7)	6	5	5
**Psychiatric syndrome + seizures/focal neurology**	19 (3.9)	4	7	5
**Peripheral neuropathy**	65 (13.2)	5	0	13
**Seizures/epilepsy**	53 (10.8)	24	42	13
**Movement disorder**	30 (6.1)	6	5	4
**Ataxic syndrome**	25 (5.1)	9	1	5
**Visual symptoms**	17 (3.7)	13	1	4
**Myelitis**	11 (2.2)	3	0	6
**Motor neuron syndromes**	10 (2.0)	2	0	3
**Other/unknown (≤ 5 patients)**	49 (10.0)	14	5	10
**Total (n, %)**	491	134 (27.2)	138 (28.1)	133 (27.0)

Abbreviations: CSF: cerebrospinal fluid; EEG: electroencephalogram; MRI: magnetic resonance imaging.

### 3.3 Supportive Investigations

268 (54.6%) patients tested for autoantibodies had ≥ 1 supportive investigation of CNS pathology (abnormal MRI, EEG, CSF). 409 (83.3%) patients had an MRI brain. 134 (27.2%) had an abnormality related to presentation. 34 (6.9%) patients had MRI features suggestive of possible AE (Graus et al. 2016). Other abnormalities in the remaining 100 patients (20.4%) were highly variable and included enhancing lesions, parenchymal signal changes, space‐occupying lesions, cortical diffusion restriction, vascular abnormalities, leukoencephalopathies, normal pressure hydrocephalus, and atrophic patterns suggestive of neurodegenerative disease. 208 (42.4%) patients underwent EEG testing, and 138 (28.1%) were abnormal. 51 (10.4%) had epileptiform discharges and/or electrographic/electroclinical seizures, including seven cases of status epilepticus. The remaining 87 (17.7%) EEG abnormalities comprised periodic discharges or slowing. 257 (52.5%) patients had CSF sampling, 133 (51.7%) of whom had a pleocytosis (> 5 cells/µl) and/or raised protein (> 0.47 mg/ml).

### 3.4 Yield and Diagnosis

Negative and positive results are summarized in table 3. In total 1.6% (34) of tests were positive in CSF and/or serum, 38.2% of which were true positives (seven NCSAA, six INAA) in 13 patients and 61.8% (21) were false positives (one NCSAA, twenty INAA) in 21 patients. The incidence of antibody‐positive AE or PNS was 0.27 cases in 100, 000 persons per year based on a catchment of 750, 000 patients. 95.2% (20) of false positives were tested in serum only. 98.4% (2127) of autoantibody tests were negative, 99.1% (2107) of which were true negatives and 0.9% (20) were false negatives. There were no discordant positive and negative results for NCSAA tested with a combination of cell‐based and tissue‐based assays (CBA and TBA) or INAA tested with a combination of TBA and neuronal line blot.

**TABLE 3:** Antibody results: antibody testing results and associated final diagnoses.

Test	Total	True positive	False positive	True negative	False negative
**NCSAA**	1683	6	1	1657	19
**No. of patients**	324	4	1	300	19
***Final Diagnosis***		NMDAR‐AIE, LGI1‐AIE	CNS lymphoma	Psychiatric, FND, dementia, delirium, peripheral neuropathy, medication effects, AIE in remission, other/unknown	Ab‐negative AE
**INAA**	478	7	20	450	1
**No. of patients**	392	4	20	367	1
***Final Diagnosis***		Anti‐Yo‐PCD, CRMP5 paraneoplastic syndrome, anti‐GAD65 encephalitis	Neuropathy, malignancy, delirium, epilepsy, IPD, CBS, CVST, medication effects	Psychiatric, FND, dementia, delirium, peripheral neuropathy, viral encephalitis, other/unknown	Ab‐negative paraneoplastic neuropathy

Abbreviations: Ab: antibody; AE: autoimmune encephalitis; CBS: corticobasal syndrome; FND: functional neurologic disorder; INAA: intracellular neuronal antigen antibody; IPD: idiopathic Parkinson's disease; NCSAA: neuronal cell surface antigen antibody; PCD: paraneoplastic cerebellar degeneration.

1683 NCSAA tests (1023 serum, 660 CSF) were sent from 324 patients. Seven positive results (0.4% of all NCSAA) returned from five patients. Six were true positives in four patients, and one was a false positive (85.7% true positives, 14.3% false positives). Two patients were diagnosed with NMDAR encephalitis (one serum and CSF positive; one CSF only) and two with LGI1 encephalitis (one serum and CSF positive; one serum only). A false positive serum NMDAR antibody occurred in the setting of CNS lymphoma. No false positive CSF NCSAA results were returned. 478 INAA tests (348 serum, 130 CSF) were sent from 392 patients. 27 positive results (5.6% of all INAA) returned from 24 patients. Seven were true positives in four patients and 20 were false positives in 20 patients (74.1% false positives, 25.9% true positives). Two were diagnosed with anti‐Yo paraneoplastic cerebellar degeneration (PCD) (both serum and CSF positive), one with anti‐CRMP5 paraneoplastic movement disorder (serum positive), and one with anti‐GAD65 encephalitis (serum and CSF positive). False positive serum INAAs included recoverin, GAD65, Zi4, amphiphysin, Hu, Yo, Sox1, Ri, and Purkinje Cell antibodies. One false positive CSF GAD65 returned in a patient with Multiple Sclerosis. True and false positive antibody cases are compared in table 4.

**TABLE 4:** Comparison of true positive and false positive antibody cases: clinical features, antibody results, and associated investigations of cases with true positive versus false positive antibodies.

	True positives (n = 8)	False positives (n = 21)
**Median age (range)**	60 (25 – 74)	68 (27 ‐ 90)
**Antibody type (n)**	NMDAR (2), LGI1 (2), GAD65 (1), Yo (2), CRMP5 (1)	GAD65 (5), Recoverin (5), Zic4 (3), Amphiphysin (2), Hu (1), Yo (1), Sox1 (1), Ri (1), Purkinje Cell (1), NMDAR (1)
**Sample type**	Paired samples sent in majority of cases (6, 75.0%); CSF positivity frequent. 6 (75.0%) CSF +ve 7 (87.5%) Serum +ve	Paired samples sent in minority of cases (6, 28.5%); CSF positivity rare. 1 (4.7%) CSF +ve 20 (95.2%) serum +ve
**Presentation features**	Focal neurology and/or seizures in majority of cases (drug‐resistant epilepsy, altered conscious state with seizures, severe ataxia, chorea)	Heterogenous presentations (isolated psychiatric or cognitive symptoms, peripheral neuropathy, myopathy, parkinsonism, seizures)
**MRI**	Performed in all cases (8, 100%), abnormal. 5 (62.5%) abnormal (LE, cerebellar FLAIR hyperintensities, multifocal FLAIR hyperintensities, unilateral MTLS)	Performed in most cases (18, 85.7%), mostly normal or heterogenous. 7 (33.3%) abnormal (ring enhancing lesions, CVST, demyelination, corticobasal degeneration)
**EEG**	Performed in majority of cases (5, 62.5%), abnormal. 4 (50.0%) abnormal (NCSE, FBDS epileptiform discharges)	Performed in minority of cases (9, 42.9%), normal. 5 (23.8%) abnormal (generalized or unilateral temporal slowing, focal seizures)
**CSF**	Performed in majority of cases (7, 87.5%), abnormal. 4 (50.0%) abnormal	Performed in minority of cases (10, 47.6%), normal 3 (14.3%) abnormal
**Final diagnosis**	AE: NMDAR, LGI1, GAD65 PNS: CRMP5, Yo‐PCD	Mimics: primary psychiatric disorder, dementia, delirium, epilepsy, MS, CNS metastases or lymphoma, IPD or PPS, medication side effects Other: McArdle's myopathy, diabetic neuropathy, idiopathic neuropathy, CVST
**Immunotherapy**	Majority (7, 87.5%)	Minority (1, 4.8%)—False positive GAD65 in MS

Abbreviations: AE: autoimmune encephalitis; CNS: central nervous system; CSF: cerebrospinal fluid; CVST: cerebral venous sinus thrombosis; EEG: electroencephalogram; FBDS: faciobrachial dystonic seizures; FLAIR: fluid attenuated inversion recovery; IPD: idiopathic parkinson's disease; LE: limbic encephalitis; MRI: magnetic resonance imaging; MS: multiple sclerosis; MTLS: mesial temporal lobe sclerosis; NCSE: non‐convulsive status epilepticus; PCD: paraneoplastic cerebellar degeneration; PPS: parkinson's plus disease.

All patients diagnosed with AE or PNS had ≥ 1 of focal neurology/seizures associated with presentation, abnormal EEG, MRI brain abnormality related to presentation, and/or CSF pleocytosis/raised protein. One patient with LGI1 encephalitis had an isolated cognitive presentation; however this was accompanied by limbic encephalitis on MRI. Five (23.8%) of 21 patients with false positive antibodies had antibody testing indicated for seizures or epilepsy, two of which (40%) had an identifiable cause (i.e., post‐stroke epilepsy, symptomatic secondary to cerebral venous sinus thrombosis).

94.1% (462) of patients were negative for NCSAA and INAA. 1676 negative NCSAA results returned from 319 patients (98.6% of all NCSAA tests). 1.1% (19) of negative tests was false negatives in 19 patients diagnosed with seronegative AE. 451 negative INAA results returned from 368 patients (94.3% of all INAA). One false negative occurred (0.2% of negative INAA) in one patient diagnosed with seronegative paraneoplastic peripheral neuropathy in the setting of metastatic lung adenocarcinoma (PNS score 6). False negative cases are summarized in table 5.

**TABLE 5:** Characteristics of false negative antibody cases: demographics, presentations, antibody results, and associated investigations of cases diagnosed with seronegative AE or PNS.

Total cases	20
**Median age (range)**	66.5 (18‐87)
**Presentation features**	Seizures, NORSE, acute‐subacute cognitive impairment, behavioral disturbances, rapidly progressive sensorimotor neuropathy
**Antibody testing**	103 tests sent. Paired samples in 60.2% of samples (62) Serum only in 34% (35) CSF only 5.8% (6)
**Supportive investigations**	Abnormal MRI 50% (10) Abnormal EEG 85% (17) Abnormal CSF 70% (14)
**Final diagnosis**	19 cases Ab‐negative possible AE 1 case paraneoplastic neuropathy (PNS score 6)
**Received immunotherapy**	95% (19)

Abbreviations: Ab: antibody; AE: autoimmune encephalitis; CSF: cerebrospinal fluid; EEG: electroencephalogram; MRI: magnetic resonance imaging; NORSE: new onset refractory status epilepticus; PNS: paraneoplastic syndrome.

2107 negative NCSAA and INAA tests returned from 442 patients without diagnoses of a seronegative AE/PNS (97.5% true negative samples). Of 442 patients with true negative tests, 26.2% (116) had an abnormal MRI, 25.6% (113) had an abnormal EEG and 25.7% (114) had an abnormal CSF. Final diagnoses were highly heterogenous. The most common were primary psychiatric disorders (16.7%, 74), peripheral neuropathy (10.2%, 45) non‐autoimmune epilepsy (7.7%, 34; 47.1% [16] lesional and 55.9% [19] non‐lesional), delirium (5.8%, 26), medication side effects (4.1%, 18), dementia (3.6%, 16), recreational drug and alcohol effects (3.6%, 16), and functional neurologic disorders (3.6%, 16). Viral encephalitis was diagnosed in five patients (1.13%), two confirmed with CSF serology (Japanese encephalitis, herpes simplex encephalitis) and three cases attributed to non‐specific viral etiologies. A further 76 final diagnoses were listed. 26 patients (5.3%) did not have a final diagnosis.

### 3.5 Management

24% (118) of patients were treated with immunosuppression, 5.9% had seropositive AE or PNS. All patients with seropositive AE and three with INAA‐associated syndromes (anti‐Yo PCD, GAD65 AE) received immunosuppression. Two cases were started only after positive antibody results returned (anti‐Yo PCD, LGI1). One patient with a false positive CSF GAD65 received immunotherapy. The remaining 94.1% (111) of patients had negative autoantibody results ‐16.1% (20) had a diagnosis of seronegative AE and 77.1% (91) had alternative diagnoses. 5 of 7 (71.4%) of treated seropositive cases (75%) were commenced on immunosuppression prior to antibody results returning. No patients had immunosuppression cease on return of negative autoantibody results.

## 4 Discussion

This single‐center study underscores the considerable variability in requests for AE and PNS autoantibody testing and low diagnostic yield. Psychiatric presentations predominated, followed by cognitive disturbances. Approximately half of cognitive presentations and over three quarters of psychiatric presentations had no associated focal neurology or seizures. Other common indications included seizures, movement disorders, ataxia, and neuropathies; however, a wide range of neurologic and non‐neurologic symptoms were identified.

The yield of true positive results was low (0.6%) despite the high number of tests sent. Eight patients were diagnosed with an antibody‐positive AE or PNS over four years with an incidence of 0.27 cases in 100, 000 persons per year. This is lower than other population studies, which reported incidence rates of 0.4–0.89 antibody‐positive cases in 100, 000 persons per year (Dubey et al. 2018, Vogrig et al. 2020). Potential factors contributing to this discrepancy include greater diversity in the racial and age demographics of the populations studied, inclusion of a broader range of antibodies, and longer observation periods (Dubey et al. 2018). The population‐based study by Vogrig et al. (2020) used the 2004 PNS diagnostic criteria (Graus et al. 2004), in contrast to the 2021 updated criteria used in our study (Graus et al. 2021). Their 9‐year follow‐up period may have also contributed to higher malignancy detection and PNS diagnosis (Vogrig et al. 2020).

All patients with true positive results had ≥ 1 of focal neurology/seizures, abnormal EEG, abnormal MRI brain, and/or CSF pleocytosis/raised protein. Key clinical features in our cohort included severe ataxia, chorea, and seizures accompanied by altered conscious state and/or drug resistance. Only one patient presented with isolated cognitive deficit without accompanying focal neurology or seizures, however, their MRI showed limbic encephalitis. MRI abnormalities in patients with antibody‐positive AE or PNS were typical of AE (limbic encephalitis, multifocal FLAIR hyperintensities) or PNS (cerebellar inflammation) (Graus et al. 2016, Madhavan et al. 2020, Hartung et al. 2024) compared with a wide spectrum of abnormalities observed in patients with false positive or truly negative results, including abnormalities explaining their presentation (i.e., CVST causing provoked seizures). Epileptiform discharges, seizures or NCSE were common EEG findings in patients with antibody‐positive AE or PNS. Of patients truly negative for autoantibodies, only one quarter had an abnormal EEG, MRI, or CSF.

No patients with isolated psychiatric presentations returned a positive autoantibody result. This is consistent with previous studies investigating the yield of autoantibody screening in first episode psychosis (FEP). Jeppesen et al. (2023) found no difference in the yield of CSF or serum autoantibodies in patients presenting with FEP compared with controls. Subsequent studies have not detected autoantibodies in patients with FEP without concurrent neurological symptoms and/or supportive investigation findings (Guasp et al. 2021, Warren et al. 2023, Chan et al. 2021). Certain features have been identified as potential “red flags” that should prompt consideration of AE in patients with psychosis, including abrupt onset of FEP accompanied by neurological symptoms (i.e., movement disorder, seizures, headache, speech disturbance), severe cognitive disturbance, reduced conscious state, dysautonomia, catatonia, increased antipsychotic sensitivity, and rapidly escalating severity, including the requirement for electroconvulsive therapy (Scott et al. 2018, Herken and Pruss 2017, Warren et al. 2020).

74.1% of positive INAA results were false positives (non‐specific). These results are comparable with previous studies reporting false positive rates between 71.3% and 78.4% (Ebright et al. 2018, Albadareen et al. 2017). The high rate of false positive INAA increases the risk of misdiagnosis, leading to inappropriate management and extensive investigations, including searching for an occult malignancy. PNS are rare (Vogrig et al. 2020, Shah et al. 2022), whereas the final diagnoses for patients with false positive results were often common disorders, such as idiopathic Parkinson's disease, delirium, lesional seizures, non‐paraneoplastic neuropathies, and medication side effects. Clinicians may inappropriately attribute the patient's neurology to a PNS based on positive antibody results and miss common, potentially reversible diagnoses.

The most frequently encountered false positive INAA were GAD65, recoverin, and Zic4. All were serum samples except for one CSF positive GAD65. GAD65 antibody titers were not provided. Serum GAD65 antibodies can be detected in 1‐ 5.9% of the general population, with a higher prevalence in individuals with diabetes mellitus and other autoimmune diseases (Lundgren et al. 2010, Ruige et al. 1997, Meinck et al. 2001). Elevated titers (> 20 nmol/L radioimmunoassay) are more specific for GAD65 antibody‐spectrum neurologic disorders (Budhram et al. 2021, Walikonis and Lennon 1998). Detection of GAD65 in CSF is strongly associated with CNS autoimmunity, (Graus et al. 2020) however, false positives have been described (Flanagan et al. 2023). Caution should be exercised when interpreting GAD65 positivity in patients with a low pre‐test probability, particularly when titers are low or unavailable. False positive serum anti‐recoverin antibodies have been reported in patients with unrelated neurologic symptoms (Vaisvilas et al. 2024). Anti‐recoverin antibodies are classically associated with cancer‐associated retinopathy (CAR) (Adamus et al. 2004, Ohguro et al. 2004). Case reports have described anti‐recoverin antibodies in AE and ataxia without retinopathy, (Kitazaki et al. 2021, Saraya et al. 2019) however, their significance in these cases appears unlikely given that recoverin is a photoreceptor protein not found in the CNS (Adamus et al. 2004, Shiraga and Adamus 2002, Matsubara et al. 1996).

Only one false positive NCSAA returned in the serum and none in the CSF. While this is a low false positive rate, the patient was diagnosed with primary CNS lymphoma, and their initial imaging showing multifocal ring enhancing lesions was not typical of NMDAR encephalitis (Zhang et al. 2018) (Kelley et al. 2017). Previous cases of CNS malignancy misdiagnosed as AE due to false positive autoantibody results have been described. Flanagan et al. (2023) found 10% of 107 patients misdiagnosed with AE had a primary CNS malignancy, including two with CSF NMDAR positivity. Testing patients without sufficient evidence of AE may delay recognition of primary CNS malignancies, resulting in worsening and potentially irreversible outcomes if treatment is delayed. Treatment of suspected AE with high dose corticosteroids (Abboud et al. 2021) may also interfere with biopsy results and impede diagnosis in cases of CNS lymphoma (Barrantes‐Freer et al. 2018).

The majority of patients tested for AE and PNS were diagnosed with common conditions, including psychiatric disorders, delirium, medication side effects, functional neurologic disorders, and dementia. These findings align with recent studies analyzing AE misdiagnosis. Flanagan et al. (2023) found functional neurological disorders, dementia, psychiatric disorders, and non‐specific cognitive syndromes to be frequent true diagnoses in cases misclassified as AE. Dementia and psychiatric diagnoses were similarly common amongst patients with false positive autoantibody results in a retrospective analysis by Vaisvilas et al. (2024).Autoantibody testing rarely influenced decisions on acute immunosuppression. Two patients were commenced on immunosuppression following the return of positive autoantibodies and none had immunosuppression ceased on the return of negative tests. Treatment decisions were primarily guided by clinical evaluation, with or without supplementary investigations, rather than by antibody test results. While autoantibody testing plays an important role in confirmation of AE and PNS, results are typically delayed and therefore cannot be used in isolation to determine treatment (Abboud et al. 2021, Irani 2024). Consideration should be given as to how testing will contribute to a patients’ diagnosis and management prior to requesting these autoantibody tests.

Our results underscore the importance of establishing the clinical syndrome prior to autoantibody testing to minimize unnecessary investigations. The sensitivity of the possible’ AE criteria is high (80– 84%) (Van Steenhoven et al. 2023, Costa et al. 2022, Li et al. 2017), and patients’ presentations should be assessed against this before considering testing (Graus et al. 2016, Van Steenhoven et al. 2023). For PNS, determining whether the patients’ presentation matches a known intermediate‐ to high‐risk phenotype can help prevent unnecessary autoantibody testing (Graus et al. 2021). Expert groups have suggested best practice guidelines to guide investigation for AE and PNS^.10, 11^Emphasis is placed on clinical phenotyping and utilization of MRI and EEG as first line tests to confirm CNS pathology suggestive of AE and exclude alternative diagnoses prior to autoantibody testing (Abboud et al. 2021). In our study, only half of patients had MRI, EEG, or CSF findings suggestive of CNS pathology, and many did not have these investigations performed. Overemphasis on rare diagnoses without supportive clinical features or investigations risks diverting focus from identifying and treating the correct underlying condition.

An autoimmune etiology is often considered in patients with new onset epilepsy of unknown cause. While timely diagnosis is important, this must be balanced against the risk of indiscriminate antibody testing in patients with a low pre‐test probability. Three (14.3%) of 21 patients with false positive autoantibody results in our cohort had non‐autoimmune epilepsy of unknown etiology. The Antibody Prevalence in Epilepsy (APE) score incorporates clinical features and paraclinical investigations to estimate the likelihood of positive autoantibodies (Dubey et al. 2017). The score has since been refined to include patients with encephalopathy (APE2) (Dubey et al. 2018). APE2 demonstrates excellent sensitivity (99%) and specificity (93%) for predicting the presence of clinically relevant autoantibodies (Dubey et al. 2018) and can serve as a valuable tool to guide testing decisions.

Future research should include prospective studies that examine the feasibility and yield of a tiered approach to antibody testing using possible AE criteria (Graus et al. 2016) and intermediate‐high risk paraneoplastic phenotypes (i.e., rapidly progressive cerebellar syndrome, sensory neuronopathy) (Graus et al. 2021) to stratify patients into low, moderate, or high pre‐test probability of AE or a PNS. This approach was recently investigated by Sharp et al. (2021) in which applying an algorithm for AE testing was associated with Increased true positive results and substantial healthcare cost savings. While the possible AE criteria are highly sensitive, their variable specificity (27–94%) (Van Steenhoven et al. 2023, Costa et al. 2022, Li et al. 2017) means they must be applied in conjunction with corroborative clinical features and investigations before making a diagnosis of AE (Van Steenhoven et al. 2023).

This study has several limitations. The retrospective design means medical records were relied on for clinical data, which may not have accurately captured the indication for testing. The moderate sample size and single‐center setting restrict the generalizability of the findings to the broader population. The absence of accurate costing data precluded evaluation of the cumulative expense and cost‐effectiveness of current testing practices. Larger, multicenter cohort studies across different health networks with integrated costing data are required to comprehensively assess patterns, diagnostic yield, and clinical impact of autoantibody testing.

The potential inclusion of non‐antibody‐mediated conditions among cases classified as false negatives is another important limitation. To address this, we defined false negative results using established clinical diagnostic criteria (Graus et al. 2016, Graus et al. 2021) to enhance the likelihood of capturing true seronegative AE and PNS cases. Seronegative status in these patients may reflect novel antibodies, or undetected antibodies due to limitations of current testing methods—constituting ‘true’ false negatives. However, alternative explanations such as non‐antibody mediated autoimmunity or diagnostic mimics must also be considered given the variable specificity of possible AE criteria (Van Steenhoven et al. 2023). ‐^58^Future research could address this issue through repeat testing in independent laboratories and examining samples for similar immunostaining patterns to facilitate the identification of novel antigens. This approach has been used by research groups previously and resulted in the identification of novel antibodies in previously “seronegative” cases, including AMPA, DDPX and GABA‐A (Lai et al. 2009, Boronat et al. 2013, Petit‐Pedrol et al. 2014). Additionally, longitudinal follow‐up of seronegative cases may provide further insight into their clinical course and help validate diagnostic accuracy.

## 5 Conclusions

AE and PNS are rare diagnoses typically associated with focal neurology/seizures and supportive radiologic, cerebrospinal fluid, and/or neurophysiologic findings. In this analysis of NCSAA and INAA, the yield of testing in patients with heterogenous indications was low and rarely altered management. False positive INAA was common. Clinical phenotyping of patients combined with supportive investigations is important to prevent inappropriate widespread autoantibody testing. Common conditions should be considered in patients presenting with psychiatric, cognitive, and neurologic presentations prior to testing for rare autoimmune diseases. Future studies focusing on requesting and testing patterns from wider regions are needed to further clarify yield and establish rigorous testing protocols that would improve accurate disease diagnosis and limit excessive utilization of healthcare resources.

## Author Contributions

**Hannah Ford**: conceptualization, methodology, data curation, investigation, formal analysis, writing – original draft. **Nabil Seery**: writing – review and editing, conceptualization. **Tracie Tan**: conceptualization, writing – review and editing. **Logan Gardner**: data curation, investigation. **Jeffrey Box**: methodology, data curation, investigation. **Robb Wesselingh**: writing – review and editing. **Julian Bosco**: data curation, writing – review and editing, conceptualization, methodology. **Mastura Monif**: conceptualization, methodology, supervision, writing – review and editing.

## Peer Review

The peer review history for this article is available at https://publons.com/publon/10.1002/brb3.70779

## Data Availability

Data is available on reasonable request from the authors.
